# Modeling the synergistic enhancement of drug permeation by a dual-microbubble system under ultrasonic excitation

**DOI:** 10.1080/10717544.2025.2597621

**Published:** 2025-12-07

**Authors:** Yingjie Li, Jiwen Hu, Yunsu Wang, Qinlin Li, Youxin Chen

**Affiliations:** a School of Mathematics and Physics, University of South China, Hengyang, People's Republic of China; b School of Electrical Engineering, University of South China, Hengyang, People's Republic of China

**Keywords:** Two-microbubble–liquid–solid system, ultrasonic excitation, vascular endothelium, cavitation, permeability

## Abstract

The high selectivity of the vascular endothelium, exemplified by the blood–brain barrier (BBB), provides critical protection to tissues against harmful substances; however, it also severely restricts the targeted delivery of therapeutic agents, particularly large molecule drugs. Ultrasound-mediated microbubble cavitation has emerged as a promising strategy for enhancing drug delivery. However, conventional single-microbubble systems suffer from limitations, including uneven energy distribution and suboptimal permeabilization efficacy. Moreover, the synergistic mechanisms underlying dual-microbubble interactions within the microvasculature remain poorly understood. In this study, we developed a coupled two-microbubble fluid–solid system (TMFSS) model utilizing the finite element method to simulate the dynamic behavior of dual microbubbles within blood vessels under ultrasonic excitation. Our investigation focused on key parameters—including microbubble spacing, acoustic pressure amplitude, microbubble size, and shear-thinning blood rheology—and their effects on microbubble oscillation, microstreaming, vascular wall stress, and endothelial permeability. The results demonstrate that, compared with single-microbubble systems, the ultrasound-assisted TMFSS significantly enhances drug permeability. This synergistic permeabilization effect strongly depends on the acoustic parameters, blood viscosity, microbubble size, and spatial distribution. Our study quantitatively elucidates the structure‒activity relationship between TMFSS dynamics and drug penetration efficiency and presents a parameter optimization strategy for the precise modulation of vascular endothelial permeability.

## 1 Introduction

The vascular endothelial barrier, particularly the blood–brain barrier (BBB), serves as a sophisticated defence system formed through biological evolution. The pore size of this barrier is strictly controlled to be less than 2 nm (Abbott et al. 2010). While this high selectivity prevents the entry of pathogens and neurotoxic substances, it also results in more than 98% of small-molecule drugs and nearly all large-molecule therapeutic agents (such as monoclonal antibodies, gene vectors, and nanoparticles) being unable to effectively reach target tissues (Pardridge 2020). Taking glioblastoma as an example, the tumour accumulation rate of systemically administered drugs is less than 0.1%, representing a critical bottleneck limiting therapeutic efficacy (Gan et al. 2017).

To overcome the limitations of the vascular endothelial barrier, researchers have developed various drug delivery technologies, including invasive direct injection (Jain 2019), hyperosmotic solution disruption (Raghav et al. 2023), and noninvasive nanocarriers (Tam et al. 2016), and receptor-mediated endocytosis (Large et al. 2019), among others. However, each of these methods has its own limitations. For example, direct injection is prone to causing brain tissue damage and infection (Ye et al. 2024). While the passive penetration of nanocarriers relies on the EPR effect (enhanced penetration and retention), their penetration depth in nontumor tissues is very limited (Kumar et al. 2021; Tian 2024). Therefore, there is an urgent need to develop novel noninvasive, high-penetration, spatiotemporally controllable delivery technologies.

The use of the ultrasound-microbubble technique, as a novel noninvasive approach, provides new opportunities for opening the BBB and enhancing drug delivery (Zhao et al. 2024). This technique utilises the cavitation effect of microbubbles (gas-filled particles typically 1–10 μm in diameter) in an ultrasonic field to mechanically and transiently increase vascular permeability, thereby promoting drug penetration across the BBB (Zhao et al. 2024; Moonen et al. 2025). Specifically, under ultrasound stimulation, microbubbles undergo periodic pulsation (stable cavitation) or violent collapse (inertial cavitation), generating shock waves, microjets, and localised microstreaming. These forces open or disrupt tight junctions between endothelial cells and enhance the convective diffusion of drug molecules (Moonen et al. 2025; Xia et al. 2025). These forces can instantly open endothelial tight junctions and increase paracellular permeability, with the closure time window controlled within 2–3 hours (Tsai et al. 2018). Compared with traditional methods, the ultrasound-microbubble technique offers several significant advantages (Zhao et al. 2024). First, this method is noninvasive and does not cause surgical trauma. Second, this method enables precise spatial and temporal control over the treatment. Third, this method causes minimal damage to normal tissues. Finally, it is compatible with a wide range of therapeutics, including small-molecule drugs, large-molecule drugs (e.g. biologics), and nanodrugs.

In actual physiological environments, microbubbles typically exist and interact in large numbers. Consequently, research on ultrasonic cavitation has evolved from single–bubble–fluid–solid coupling models to multiple–bubble–fluid–solid coupling models, aiming to elucidate the mechanical mechanisms underlying ultrasound-enhanced drug delivery. Among these models, the two-bubble system results in synergistic cavitation behaviour within microvessels. This behaviour offers valuable insights into the drug transport efficiency and biological effects of multiple microbubble systems (Chowdhury et al. 2017). To characterise cavitation dynamics under dual-microbubble interactions, researchers have developed coupled vibration models for bubble pairs, extending the classical Rayleigh‒Plesset equation (Xu and Wang 2023; Wang et al. 2023; Doinikov and Bouakaz 2015; Lei et al. 2024). Typical models include the coupled equations proposed by Doinikov and Bouakaz (Doinikov and Bouakaz 2015), which simultaneously account for the radial oscillations and translational movements of the bubble pair. These models reveal the secondary Bjerknes force acting between bubbles (Wang et al. 2023; Lei et al. 2024). For instance, classical linear theory predicts mutual repulsion when the driving frequency lies between the resonance frequencies of the two bubbles and attraction otherwise (Wang et al. 2023). This frequency-dependent behaviour has been further confirmed by more complex models and numerical simulations. Together with factors such as interbubble distance and size disparity, it affects the magnitude and direction of the microbubble interaction forces (Wang et al. 2023; Lei et al. 2024; Qin et al. 2021).

Comparative analysis of the wall shear stress induced by single and paired microbubbles oscillating within blood vessels reveals that, under identical conditions, simultaneous oscillation of two microbubbles reduces the oscillation amplitude of each individual microbubble. Nevertheless, the aggregate shear stress exerted on the vascular wall exceeds that produced by a single oscillating microbubble (Guo et al. 2024). Yuan et al. experimentally discovered that the high-speed counterflow jets generated by the collapse of two bubbles collide and converge, resulting in the formation of strong turbulence and shear forces. These forces can induce significant concave deformation in adjacent cell membranes and create larger pores than those produced by a single bubble, thereby increasing the efficiency of macromolecular uptake (Yuan et al. 2015). Compared with a single bubble, the jet flow field generated by dual bubbles is more complex and intense and is capable of instantly inducing a larger area of cellular damage or permeability enhancement (Yuan et al. 2015). Therefore, mechanistically, the interaction within a dual-bubble system can generate stronger turbulence and shear forces than a single bubble can, with significant implications for the sonoporation of endothelial cells (Wang et al. 2023; Lei et al. 2024). Additionally, exciting microbubbles of different sizes to resonance may enable safer and more efficient drug delivery (Lei et al. 2024; Xu et al. 2018). However, most current in vivo experiments rely on empirical parameters and lack systematic investigations into the optimal parameters governing dual-microbubble dynamics.

Although two-bubble systems have shown potential for enhancing drug delivery in recent years, many challenges remain to be addressed. In confined environments such as microvessels, the cavitation behaviour of a bubble pair exhibits distinct kinetic characteristics compared with those of unbounded scenarios. Near–wall effects and bubble–bubble coupling jointly influence cavitation patterns. To align with experimental data, simulations require the incorporation of additional realistic factors, such as the constraining effects of vascular walls, surrounding flow fields, and tissue influences (Chowdhury et al. 2017). Existing theoretical studies employ various mathematical models—such as microbubble dynamics models, microbubble–vascular wall interaction models, drug transport/diffusion models, and multiphysics coupling models—to describe this process. However, these models often involve numerous assumptions and simplifications, making it difficult to fully capture real physiological conditions. For example, blood is frequently modelled as a Newtonian fluid (Wang et al. 2023; Lei et al. 2024; Doinikov and Bouakaz 2015). Actually, the oscillatory behaviour of dual microbubbles within blood vessels is typically nonlinear, inducing complex fluid mixing. Importantly, under high-acoustic-pressure or high-frequency conditions, blood viscosity exhibits shear-thinning behaviour, which varies with the fluid shear rate (Kim et al. 2017; Wu et al. 2018; Gijsen et al. 1999). Building upon established microbubble dynamics models, this study introduces an enhanced two-microbubble fluid–solid system (TMFSS). The key advancement lies in integrating shear-rate-dependent blood viscosity into the TMFSS model, enabling a more precise characterisation of blood's non-Newtonian rheology under ultrasonic cavitation. This refinement facilitates more physiologically realistic simulations of hemodynamics and vessel wall mechanical responses. Using this improved model, we systematically investigated the influence of ultrasound parameters (pressure and frequency), microbubble properties (size and spacing), and their interactions on microbubble oscillation patterns, vascular wall stress distribution, and endothelial permeability enhancement.

## 2 Theoretical models and methods

### 2.1 Geometric model

**Figure 1.**
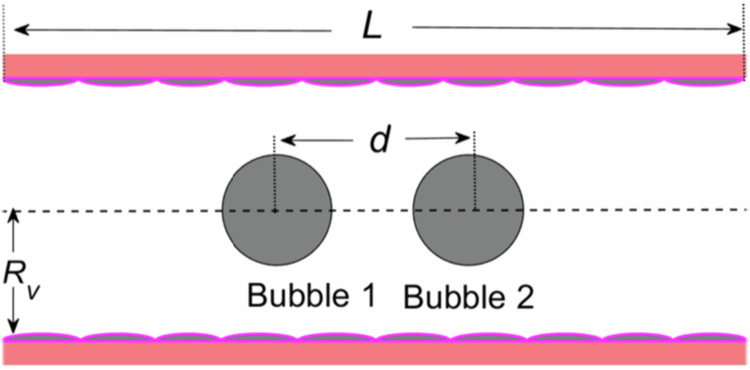
Geometric sketch of a two-dimensional microbubble-vessel system.

The TMFSS coupling model is shown in Figure 1. In a straight segment of a blood vessel with a length of L=40μm, two shell-free microbubbles are located along the central axis of the vessel, with radii $R_{1}$ and $R_{2}$ and a distance d between their centres. The vessel radius is denoted as $R_{V}$ and set to 5 μm. It is assumed that the vessel wall consists of a porous elastic material, with the inner surface of the vessel lined by endothelial cells that have gaps between them. In Figure 1, the pinkish-red, nonuniform protrusions represent the vascular endothelium. Since this study mainly focuses on the permeability by regulation of the endothelial gap under steady-state cavitation, the applied driving acoustic pressure is in the order of tens of kilopascals, and the excitation frequency is in the megahertz range.

### 2.2 Hemodynamic model

Considering blood as a non-Newtonian fluid, we employed the Carreau‒Yasuda model to characterise the hemodynamics of blood vessels (Figure 1) (Gijsen et al. 1999a; Gijsen et al. 1999b). Under the assumption of incompressible blood flow with velocity field u and pressure field p, the governing equations consist of the continuity equation and momentum conservation equation, expressed as follows:

(1) ∇∙u=0

(2) $$\rho_{f}\left(\frac{\partial u}{\partial t}+u∙\nabla u\right)=-\nabla p+\nabla ∙\tau_{f}$$

where $\rho_{f}$ is the blood density and $\tau_{f}$ is the blood viscosity stress tensor, whose divergence term is as follows:

(3) $$\nabla ∙\tau_{f}=\nabla ∙\left(2\eta \left(\dot{\gamma }\right)D\left(u\right)\right)=2\nabla ∙\left(\eta \left(\dot{\gamma }\right)D\left(u\right)\right)$$

where $\eta (\;\dot{\gamma }\;)$ denotes the shear rate-dependent dynamic viscosity and D(u)is the rate of deformation tensor, which is given as follows:

(4) $$D\left(u\right)=\frac{1}{2}(\nabla u+({\nabla u)}^{T})$$

The second invariant of the rate of deformation tensor D must be used to define the shear-rate parameter *γ*, which is expressed as follows for incompressible fluids:

(5) $$\dot{\gamma }=\sqrt{2D_{v}∶D_{v}}$$

The Carreau–Yasuda model provides an excellent description of the non-Newtonian behaviour of blood (Gijsen et al. 1999) and is written as follows:

(6) $$\eta \left(\dot{\gamma }\right)=\eta_{\infty }+\left(\eta_{0}-\eta_{\infty }\right){\left[1+{\left(\lambda \dot{\gamma }\right)}^{\alpha }\right]}^{\frac{n-1}{a}}$$

Equation (6) contains five parameters. Their meanings and corresponding values are listed in Table 1.

**Table 1.** Blood model parameters in this study (Pereira et al. 2013; Abugattas et al. 2020; Johnston et al. 2024).

Name	Parameters	Value (unit)
Zero shear rate viscosity	$\mu_{0}$	0.0519 Pa∙s
Infinite shear rate viscosity	$\mu_{\text{∞}}$	0.00476 Pa∙s
Relaxation time	λ	0.438 s
Power index	n	0.191
Transition parameters	a	0.409

### 2.3 Bubble dynamics model

If the gas inside the bubble satisfies the ideal gas equation of state, the heat transfer during bubble oscillation is neglected.

(7) $$p_{g}=p_{g0}{\left(\frac{R_{i0}^{3}}{R_{i}^{3}}\right)}^{\kappa }$$

(8) $$p_{g0}=p_{0}+\frac{2\sigma_{g}}{R_{i0}}-p_{v}$$

where $p_{g}$ and $p_{g0}$ represent the gas pressure inside the i-th bubble at time t and the initial gas pressure, respectively. $R_{i}\;$and $R_{i0}$ represent the bubble radius of the i-th bubble at time t and the initial radius, respectively. k is the polytropic exponent of gas, and $p_{0}$ and $p_{v}$ are the initial static pressure in the liquid and the vapour pressure inside the bubble, respectively.

Under ultrasonic excitation, one obtains the following at the bubble–liquid interface:

(9) $$((p_{g}-p_{0})I+\mu (\dot{\gamma )}(\nabla \cdot u+\nabla \cdot u^{T}))\cdot \vec{n}=2\sigma k_{R}n$$

Here, I, n*,* and $k_{R}$ are the identity tensor, unit normal vector, and local curvature of the bubble surface, respectively.

In a system consisting of two microbubbles, the interaction force between the microbubble-es, conventionally called the secondary Bjerknes force, $F_{\mathrm{SB}},\;$, can be expressed as follows:

(10) $$F_{\mathrm{SB}}=-\frac{\rho_{f}}{4\pi d^{2}}{\dot{V}}_{1}{\dot{V}}_{2}$$

where $V_{1}$ and $V_{2}$ represent the volumes of bubble 1 and bubble 2, respectively, with the dots above them denoting the time derivatives of the volumes.

### 2.4 Solid models and fluid–solid

Assuming that the vessel wall is a uniform, isotropic, linear elastic material and neglecting its volume forces, the dynamic process of the vessel wall over time is momentum conservation given by:

(11) $$\rho_{s}\frac{\partial^{2}d}{\partial t^{2}}=\nabla ∙\sigma_{s}$$

where $\rho_{s}$ is the vessel wall density and ${\text{σ}}_{s}$ is the solid stress tensor.

### 2.5 Pharmacokinetic model

If the shear stress on the vascular wall consists of two components, one being the shear stress generated by ultrasonic cavitation and the other originating from the mechanical properties of the cells themselves. The total shear stress can be expressed as follows:

(12) $$\tau =\tau_{\mathrm{ex}}-E\varepsilon$$

where E is the Young's modulus and ε is the strain. Variable $\tau_{e}$ represents the shear stress generated by microstreaming on the wall surface under the drive of microbubbles and is expressed as $\tau_{\mathrm{ex}}=\eta (\dot{\gamma })\frac{d{\text{u}}_{w}}{\mathrm{dr}}\;\;{⃒}_{w}$, where $u_{w}=\sqrt{u^{2}+v^{2}}$ in the *zr*-plane.

Considering the relationship between shear stress and endothelial permeability (Himburg et al. 2004), as well as experimental reports (Olgac et al. 2008), gaps between adjacent endothelial cells undergo deformation under the shear stress induced by microstreaming, and the deformation factor can be expressed by the following equation (Olgac et al. 2008):

(13) $$\mathrm{SI}=0.38\times e^{-0.79\cdot \tau }+0.225\times e^{-0.043\cdot \tau }$$

Based on previous work (Olgac et al. 2008), the permeability generated under the action of shear stress (Equation 7) can be expressed as follows:

(14) $$K=\frac{4\times 10^{8}}{3}\pi R_{\mathrm{cell}}w^{3}(0.479+0.00593e^{-14.75*\mathrm{SI}})$$

where w is the half-width of the leaky junction and $R_{\mathrm{cell}}$ is the radius of the endothelial cell.

### 2.6 Boundary conditions and initial conditions

In the bubble–fluid–solid system, two interaction boundaries between the fluid–solid and bubble–fluid should be built. The continuity principle is met in the bubble–fluid interaction.

(15) $$\dot{R}=u_{f}$$

where $u_{f}$ is the fluid velocity of the microbubble wall and $\dot{R}$ is the velocity of the microbubble wall. Similarly, in the process of blood and vessel wall action, the continuity of velocity and force is satisfied; the following equation can be obtained:

(16) $$u_{s}=\frac{\partial d_{s}}{\partial t}$$

(17) $$\sigma ∙n=\left(-pI+\mu (\dot{\gamma }\right)(\nabla u_{s}+{\left(\nabla {\text{u}}_{s}\right)}^{T}∙n$$

where $u_{s}$ and n are the vibration velocity of the vessel wall and the unit normal phase vector, respectively.

The initial conditions are set as follows:

(18) $$u\left(x,0\right)=0,\;p\left(x,0\right)=p_{0},\;d\left(x,0\right)=0,\;\partial_{t}d\left(x,0\right)=0$$

### 2.7 Numerical simulation

In our study, we utilised the finite element method through COMSOL Multiphysics 5.5 (COMSOL, Inc.; Burlington, MA) to solve the equations governing TWFSS interactions along with their initial and boundary conditions. As illustrated in Figure 1, a unit-size free triangle mesh was employed to partition the model. Given the dynamic nature of the bubble–fluid boundaries, we implemented an arbitrary Lagrangian–Eulerian approach to adeptly manage the moving meshes. Moreover, the concave walls were modelled using a moving mesh methodology. To optimise the computation time, we considered the system to be two-dimensional axisymmetric and subsequently decomposed it into a fluid domain and a wall domain. For grid generation, a maximum mesh size of λ/6 (where λ represents the wavelength) was adopted in the focal region, whereas a maximum mesh size of λ/4 was used in the remaining part of the domain. The vessel walls were composed of 5,086 vertices of mesh and 9,570 triangular elements, the plaque was constructed with 710 vertices and 1,208 triangular elements, and the blood contained 4,937 vertices and 9,570 triangular units.

Unless otherwise specified, the parameters used in the model calculations are primarily those listed in Table 2.

**Table 2.** Values of parameters in this study (Lei et al. 2024; Khanafer and Berguer 2009; Qin and Ferrara 2007).

Name	Parameters	Value (unit)
Gas polytropic	γ	1.07
Saturated vapour pressure	$P_{v}$	2330 Pa
The initial radius of the microbubble	$R_{0}$	2 μm
Gas-liquid surface tension	$\sigma_{g}$	0.056 $N∙m^{-1}$
The density of blood	ρ	1059 $\mathrm{Kg}∙m^{-3}$
Sound pressure	$P_{d}$	0.3 × 10^5^ Pa
Hemodynamic viscosity	$\mu_{f}$	0.0035 Pa∙s
The ambient pressure	$P_{0}$	1.013 × 10^5^ Pa
Blood vessel density	${\text{ρ}}_{\text{w}}$	1049 $\mathrm{Kg}∙m^{-3}$
Ultrasonic frequency	f	1.5 × 10^6^ Hz
Young's modulus	E	4 × 10^6^ Pa
Vascular Poisson's ratio	ν	0.49

## 3 Results

### 3.1 Model validation

**Figure 2.**
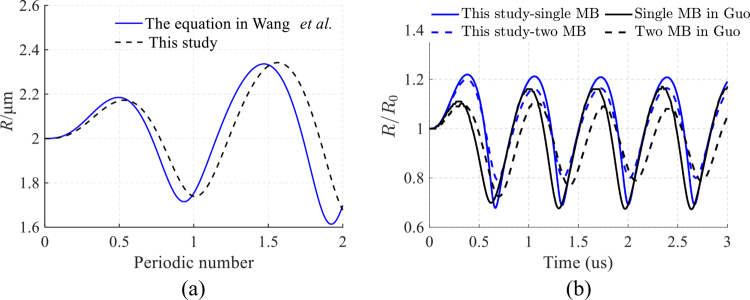
Bubble radius‒time curves for different models. the model from (Wang et al. 2022) is shown in (A), with the main parameters set as follows: a driving acoustic pressure of 0.3 × 10⁵ Pa, a driving frequency of 1.5 MHz, and an interbubble distance of 10 μm. the Model from (Guo et al. 2024) along with its main parameters are presented in (B).

In this section, we compare the dynamics of microbubbles under two different conditions. A comparison of the microbubble radius R as a function of cycle number is shown in Figure 2a. The periodicity and amplitude of the radius oscillations are in good agreement between the two curves, although our model exhibits a slight phase advance ‘drift’. This discrepancy may arise from the different computational methods used in the two models. The normalised bubble radius $R/R_{0}$ versus time for two scenarios—a single microbubble (MB) and two microbubbles—are compared in Figure 2b. Both studies show strong agreement in terms of oscillation frequency and phase, but our results predict slightly larger oscillation amplitudes and less damping. The presence of two microbubbles reduces the oscillation amplitude in both datasets, but the reduction is more pronounced in Guo’s results (Guo et al. 2024). The results shown in Figure 2 demonstrate that our computational model and methodology are acceptable and effective.

### 3.2 Dynamics of a single microbubble and two microbubbles

**Figure 3.**
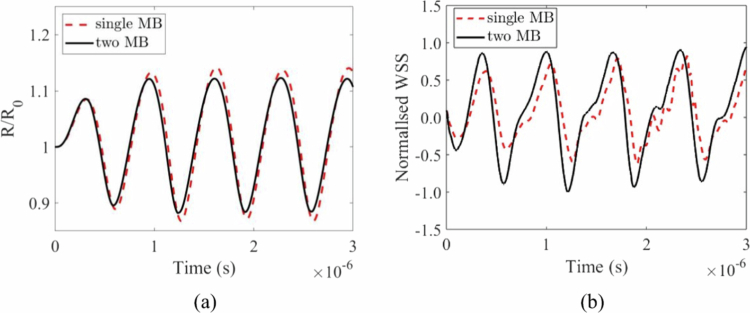
Cavitation dynamics under ultrasonic excitation. (a) normalised bubble radius versus time. (b)vessel wall normal stress versus time.

As shown in Figure 3, the dynamic behaviour of a single microbubble–fluid–solid system (SMFSS) and a coupled TMFSS model is demonstrated under ultrasonic excitation. After normalisation, the microbubble vibrations and the stresses they exert on the vessel wall in both models remain relatively stable over time. The amplitude of single microbubble oscillations is greater than that of double microbubbles, whereas the stress generated on the wall is lower than that of double microbubbles. This finding indicates that the coupling effect of double microbubbles is quite significant. These results are highly consistent with the computational results of the model (Guo et al. 2024) in terms of the trends of key variables, thereby validating the effectiveness of this model.

Notably, the results in the literature (Guo et al. 2024) are based on albumin-shelled MBs and blood flow with a constant viscosity coefficient, whereas this model is based on the Carreau–Yasuda blood flow model. Therefore, the stress on the wall exhibits distinct nonlinear characteristics, which is consistent with the model's inherent logical consistency.

**Figure 4.**
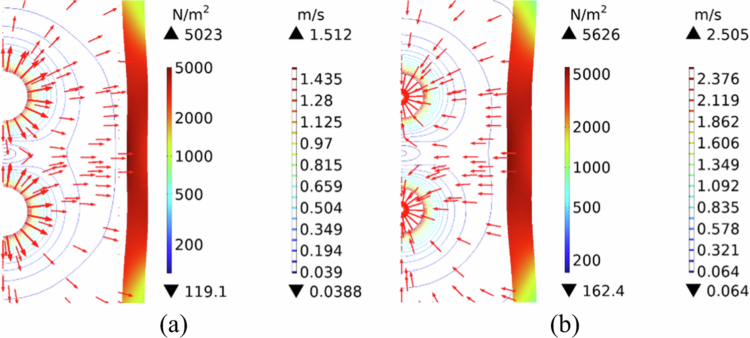
distribution of microstreaming in blood vessels and the stress generated on the vessel wall. (a) expansion phase. (b) contraction phase.

Figure 4 illustrates the microstreaming distribution and stress distribution on the vascular wall during the phase of maximum expansion (Figure 4a) and the phase of minimum contraction (Figure 4b). The maximum microstreaming and stress on the vascular wall during microbubble contraction are greater than those during expansion. Microbubble shrinkage usually leads to vessel invagination (Liu et al. 2021; Chen et al. 2010). Similar conclusions have been drawn in both theoretical and experimental studies of single microbubbles (Liu et al. 2021; Chen et al. 2010; Chen et al. 2011). These findings indicate that microbubble contraction is more likely to cause vascular damage.

### 3.3 Synergistic effect of two microbubbles

**Figure 5.**
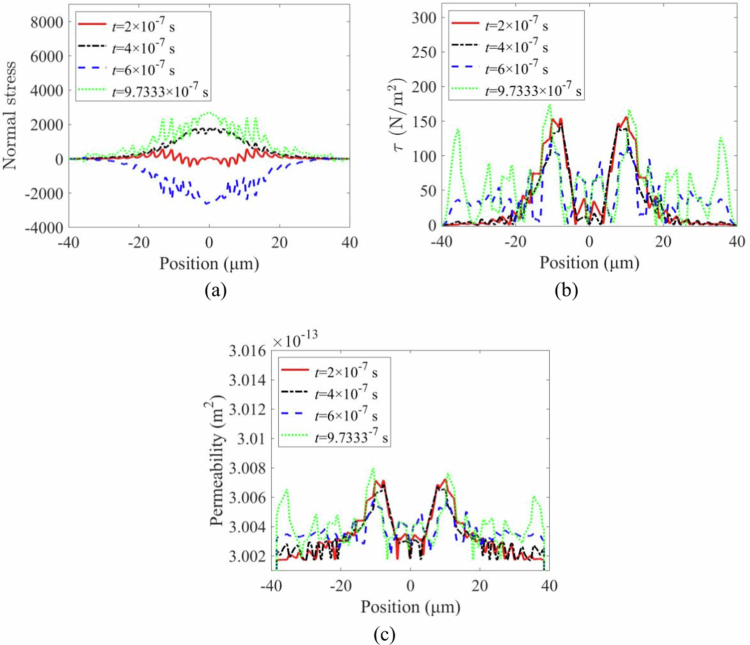
Stress and permeability distribution in the vessel wall at different moments in time: (a) normal stress variation with position on the vascular wall surface. (b)shear stress variation with position on the vascular wall surface. (c) permeability variation with position on the vascular wall surface.

The spatial distributions of stress and permeability on the vascular wall at different times induced by microbubble-induced microflows are shown in Figure 5. The normal stress resulting from direct impingement on the vascular wall by microbubbles (Figure 5a) significantly exceeds the shear stress generated by microstreaming along the wall surface (Figure 5b). Under relatively mild ultrasonic conditions ($p_{0}$ = 50 kPa), nonlinear deformation of microbubbles can induce relatively stable microstreaming (Cattaneo 2025). The wall stresses (including both normal and shear components) exhibit distinct oscillatory characteristics at different time points—a stress feature absent in single-bubble scenarios (Liu et al. 2021). Figure 5b further shows that while stresses are concentrated primarily near the dual-bubble regions, high-frequency oscillations emerge at t = 9.7333 × 10^−7^ s, manifested as increased stress peaks with reduced individual peak intensity. This phenomenon likely stems from complex vortex flows generated by oscillating dual bubbles, particularly near the vascular wall. Consequently, the wall stresses induced by dual bubbles inevitably lead to intricate permeability patterns (Figure 5c). Notably, within confined microvessels, cavitation-induced stresses predominantly manifest as normal stresses, with greater magnitudes occurring during vascular invagination than during dilation. These findings align with previously reported theoretical and experimental results (Chen et al. 2011; Xie et al. 2022).

### 3.4 Blood viscosity effect

**Figure 6:**
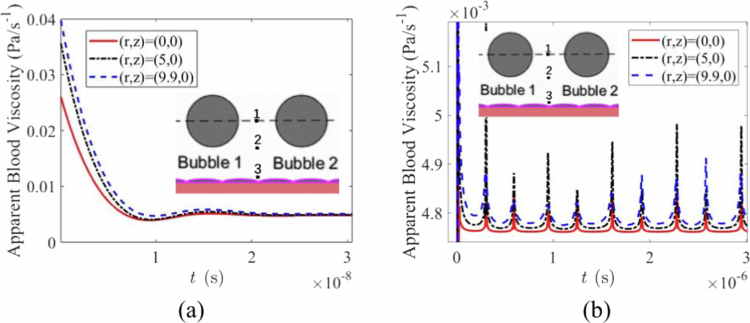
Curve of blood viscosity over time. (a) apparent blood viscosity at three observation positions (1–3). (b) magnified view of the initial stage of the apparent blood viscosity evolution shown in (a).

**Figure 7.**
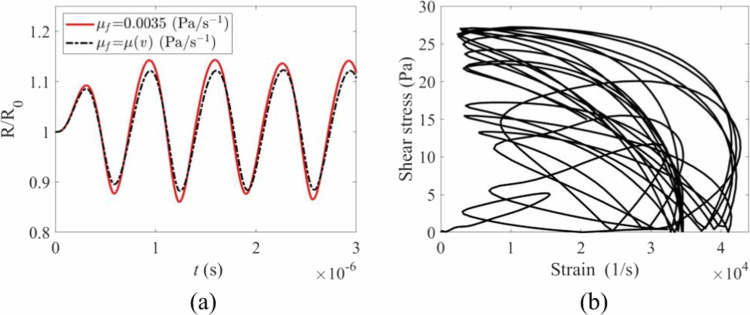
Mechanical effects of rheological blood viscosity. (a)radius variation with time under different viscosity coefficients. (b)shear stress variation with strain.

The blood flow viscosity coefficient depends on the blood flow shear rate. The viscosity coefficient at three locations in the blood flow field as a function of time is shown in Figure 6. The viscosity coefficient varies with flow velocity at different locations, with relatively large changes in the viscosity coefficient near the blood vessels. Changes in the viscosity coefficient affect microbubble dynamics because viscosity dissipation in the surrounding fluid causes energy loss, which dampens the microbubble system. The effects of the viscosity coefficient being constant and the Carreau–Yasuda model on the microbubble oscillation are shown in Figure 7a. This finding indicates that the oscillation of microbubbles within blood vessels has more complex effects on the surrounding environment.

In fact, the shear stress as a function of the strain rate in Figure 7b clearly demonstrates this complex and variable characteristic. Overall, the shear stress initially increases gradually with increasing shear rate until it reaches a peak value, after which it gradually decreases. In the low shear rate region, the curve has a relatively gentle slope, indicating that the increase in shear stress is slow under low shear rates. In contrast, the high shear rate region shows significant fluctuations in the curve, suggesting that the material’s rheological behaviour becomes complex and unstable at high shear rates, with pronounced variations in shear stress. Notably, the curve displays a distinct peak, which represents a critical point in the shear stress‒shear rate relationship, reflecting the material's maximum load-bearing capacity at a specific shear rate; indeed, owing to the diversity of linking structures between endothelial cells (Liu 2010), the stress‒strain response is nonlinear even if the external force applied varies linearly (Esfahani 2021). This highly nonlinear relationship suggests that before vascular endothelial cells are damaged, the pores between cells can open and close repeatedly.

### 3.5 Effects of blood viscosity on SMFSS and TMFSS

**Figure 8.**
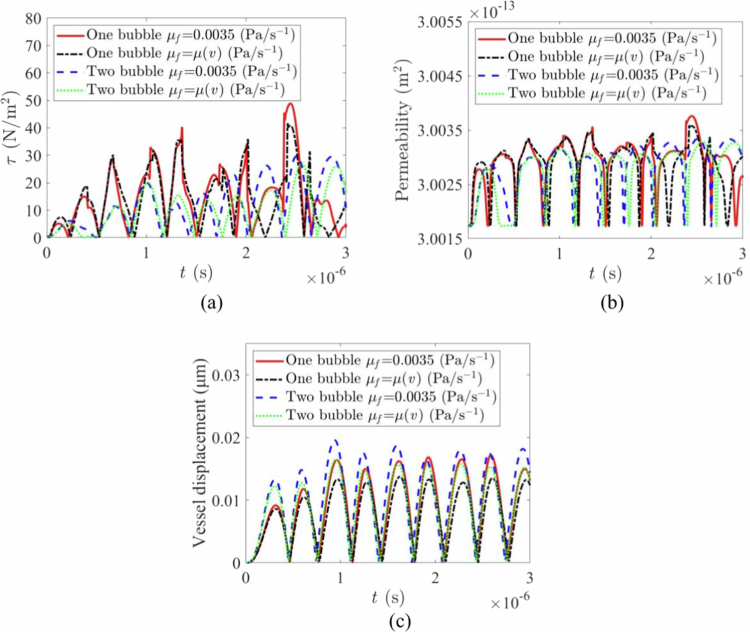
Mechanical effects under different vibration systems: (a) shear stress variation with time under different blood rheological viscosity coefficients. (b) permeability variation with time under different blood rheological viscosity coefficients. (c) vessel displacement variation with time under different blood rheological viscosity coefficients.

To further investigate the synergistic effects produced by microbubbles, a point (r = 10, z = 0) on the wall was selected to observe the kinetic effects produced by a single microbubble and two microbubbles at that point. As shown in Figure 8, when a single microbubble has a constant viscosity coefficient, the maximum shear stress is less than 20 Pa. However, in a blood flow environment with a time-varying viscosity coefficient, the shear stress acting on the wall surface is even lower. Compared with that under single microbubble oscillation, the amplitude of the shear stress curve under double microbubble oscillation is generally greater, and the curve changes are more complex. Specifically, under variable viscosity coefficient conditions, the combined effects of double-bubble interactions and variable viscosity cause the fluid viscosity to change with velocity, thereby altering the flow field around the double bubbles. The relative motion between the two bubbles and flow field interference is further complicated by viscosity feedback, resulting in stress changes that exhibit high nonlinearity in amplitude, frequency, and patterns; this reflects the complex mechanical response of multibubble systems in variable viscosity fluid environments under multiphysics coupling (fluid‒solid coupling and viscosity‒velocity coupling).

The difference in shear stress amplitude under the two oscillation modes is primarily illustrated in Figure 8a, whereas the difference in the number of permeability pulses (the number of permeation events per unit time) is highlighted in Figure 8b. As shown in Figure 8b, regardless of whether the viscosity coefficient is constant or time-varying, the permeability curve amplitude remains relatively stable under dual microbubble oscillation system conditions, and the number of permeability pulses is significantly greater than that under single microbubble oscillation conditions. This finding reflects that in the dual microbubble oscillation system, owing to the coupled synergistic effects between microbubbles, blood flow, and vessels, the permeability efficiency of vessels can be greatly enhanced. The vascular oscillation displacement curve in Figure 8c also demonstrates the synergistic effect of the dual microbubble oscillation system.

### 3.6 Effect of driving acoustic pressure on biomechanical loading and permeability

**Figure 9.**
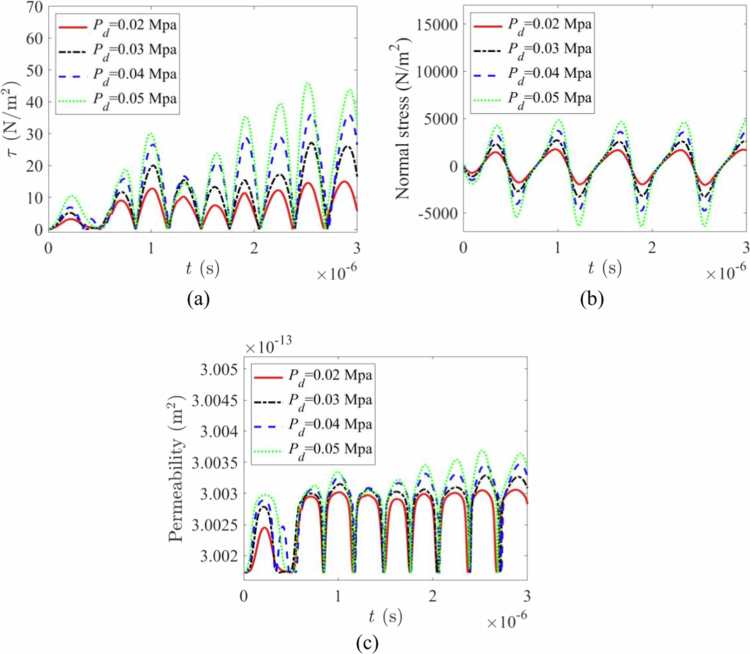
Effects of cavitation at different driving pressures. (a) shear stress variation with time under different sound pressure amplitudes. (b) normal stress variation with time under different sound pressure amplitudes. (c) permeability variation with time under different sound pressure amplitudes.

The mechanical effects on the vessel wall under different acoustic driving pressures (50, 100, and 150 kPa) are shown in Figure 9. As expected, both the shear stress (Figure 9a) and normal stress (Figure 9b) on the vessel wall increased significantly with higher driving pressures. Notably, the stress during vessel invagination (negative normal stress) was substantially greater than during distention, indicating a higher risk of vessel rupture during the contraction phase (Chen et al. 2010; Chen et al. 2011). The associated vascular permeability also increased with pressure (Figure 9c). However, the results demonstrate that the TMFSS, due to its synergistic effect, can achieve a significant permeabilization effect even at a lower pressure (e.g. 50 kPa), which helps protect blood vessels from damage.

### 3.7 Effect of the driving frequency

**Figure 10.**
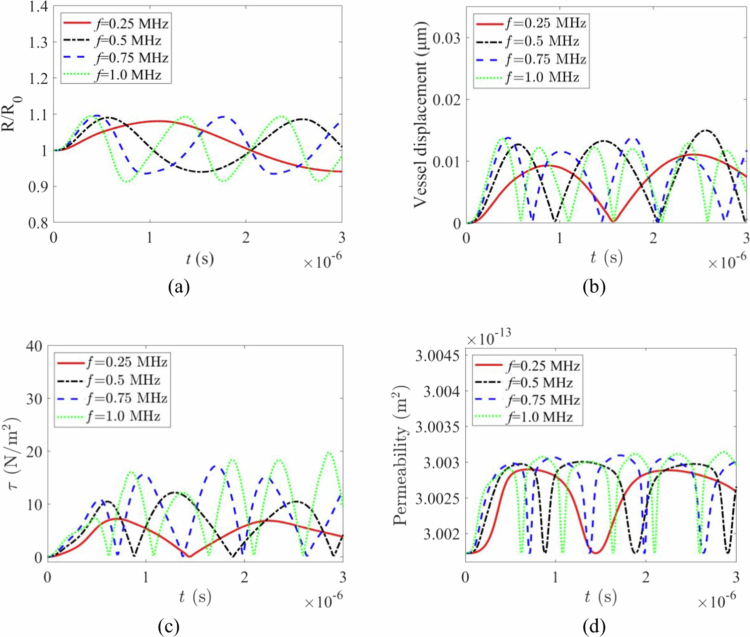
Effects of cavitation at different driving frequencie. (a) normalised bubble radius with time under different frequencies. (b) vessel wall displacement with time under different frequencies. (c) shear stress with time under different frequencies. (d) permeability with time under different frequencies.

The effects of cavitation under various acoustic driving frequencies (0.25, 0.5, 0.75, and 1.0 MHz) are shown in Figure 10. The normalised radial oscillation significantly amplifies as the driving frequency approaches the intrinsic frequency of the microbubbles, while the amplitude decreases when deviating from this frequency, indicating a ‘peak-shaped’ distribution of cavitation intensity (Figure 10a). The displacement of the vessel wall is highly correlated with bubble volume oscillations, increasing with enhanced oscillations. In the high-frequency range, the response weakens due to the dominance of fluid-wall inertia (Figure 10b). Shear stress is related to the rate of radius change and the adjacent flow velocity gradient, reaching a peak when the product of amplitude and frequency is large (near resonance). At too low frequencies, insufficient acceleration, and at too high frequencies, minimal displacement, both lead to a reduction in peak shear stress (Figure 10c). The increase in permeability is driven by both wall mechanical deformation and transient shear, with an overall positive correlation to (Figure 10a–c), achieving the maximum permeability enhancement near resonance and significantly decreasing when far from resonance (Figure 10d). Figure 10 reveals the frequency sensitivity of cavitation in the dual-microbubble-vascular system.

### 3.8 Influence of the microbubble size

**Figure 11.**
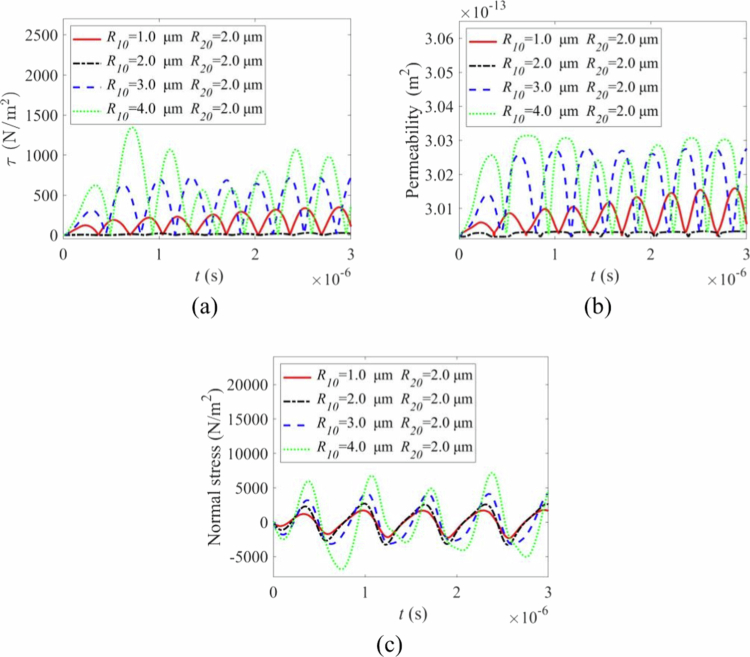
Effect of cavitation for different combinations of initial microbubble radii. (a) shear stress variation with time under varying $R_{10}$ parameters. (b) permeability variation with time under varying $R_{10}\;$parameters. (c) normal stress variation with time under varying $R_{10}$ parameters.

We investigated the effect of asymmetric bubble sizes by varying the initial radius $R_{10}$ of one microbubble (1, 2, 3, and 4 μm) while keeping the other fixed at 2 μm. The results are shown in Figure 11. As $R_{10}$ increased, the amplitudes of both the shear stress (Figure 11a) and the resulting permeability oscillations (Figure 11b) increased. The normal stress also followed this trend (Figure 11c). This is attributed to the larger bubble dominating the oscillation dynamics in the coupled system. High shear stress events (on the order of a few kPa) correlated strongly with sharp increases in permeability, confirming that mechanical forces from cavitating microbubbles directly facilitate transport across the vessel wall (Helfield et al. 2016).

### 3.9 Effect of inter-bubble spacing

**Figure 12.**
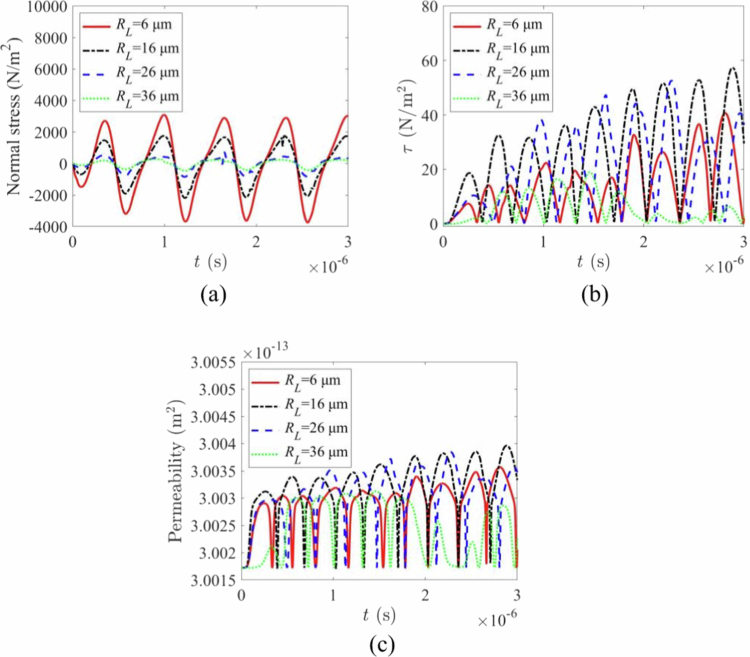
Effect of interbubble distance on microbubble-induced biomechanical effects. (a)normal stress variation with time under different bubble-bubble distances. (b)shear stress variation with time under different bubble-bubble distances. (c)permeability variation with time under different bubble-bubble distances.

The influence of the centre-to-centre distance d between the two microbubbles is shown in Figure 12. As the spacing d increased, the normal stress on the vascular wall decreased monotonically (Figure 12a). In contrast, the shear stress (Figure 12b) and the resulting vascular permeability (Figure 12c) initially increased, reaching a maximum at an intermediate distance (e.g. d=26μm), before decreasing at larger distances (e.g. d=36μm). This non-monotonic behaviour indicates that there exists an optimal separation where microbubble interactions create the most effective microstreaming and shear forces on the endothelium (Lei et al. 2024; Schutt et al. 2014). At excessively large distances, the bubbles oscillate nearly independently, and their synergistic effect diminishes.

## 4 Discussion

We have described the main characteristics of the synergistic effects observed in the dual microbubble oscillation mode, which have been addressed rarely in previous studies (Jiao et al. 2015; Liu et al. 2024; Lei et al. 2024; Qin et al. 2021). The primary finding is that the TMFSS generates superior permeabilization effects compared to the SMFSS, primarily through amplified and complex mechanical stresses on the vascular wall. This enhancement is governed by an interplay of acoustic parameters, microbubble configuration, and the non-Newtonian rheology of blood.

The key advantage of the TMFSS lies in the coupled oscillations of the two microbubbles. Our results demonstrate that this coupling leads to more complex stress patterns on the vessel wall, including high-frequency oscillations absent in single-bubble scenarios (Figure 5). This phenomenon likely stems from complex vortex flows and constructive interference of pressure waves generated by the oscillating bubble pair. The resulting biomechanical environment features not only higher peak stresses but also a higher frequency of stress/permeability pulses (Figure 8b), which collectively enhance paracellular transport efficiency. The finding that shear stress and permeability exhibit a non-monotonic relationship with interbubble distance (Figure 12) further underscores the importance of synergistic interactions, which are maximised at a specific, optimal separation (Schutt et al. 2014; Lei et al. 2024).

Our model validates that the oscillation amplitude of a single microbubble is greater than that of a microbubble in a pair (Figure 3a), consistent with the damping effect of bubble-bubble interactions (Guo et al. 2024). However, contrary to what might be expected from this damping, the TMFSS produces significantly higher wall stresses (Figure 3b). This apparent paradox highlights that the enhanced bioeffect is not due to individual bubble amplitude, but to the collective fluid dynamics and direct mechanical coupling between the bubbles and the vessel structure. The predominance of normal stress over shear stress (Figure 5) and the greater stress magnitude during invagination align well with previous theoretical and experimental studies (Chen et al. 2011; Xie et al. 2022), confirming the robustness of our model.

Grounded in bubble–vessel coupling, BBB opening exhibits an ‘optimal near-resonance under control’ behaviour: larger microbubble oscillations amplify vessel-wall displacement and shear, transiently increasing permeability, whereas overly high frequencies raise thermal dose and injury risk. In practice, transcranial focused ultrasound (FUS) with microbubbles typically employs lower frequencies of ~0.25–1.0 MHz to mitigate skull absorption and scattering (Cammalleri et al. 2020; Pouliopoulos et al. 2020). By contrast, implantable or intraoperative acoustic-window systems (e.g. SonoCloud−9) commonly use >1 MHz (≈1–1.05 MHz), enabling focal, repeatable openings that have shown reversible, safe BBB modulation and enhanced drug delivery in studies of recurrent glioblastoma and Alzheimer’s disease (Carpentier 2024). It should be noted that the frequency selection also needs to be collaboratively optimised with the pressure threshold, pulse sequence, microbubble dose, etc. Compared with the SMFSS model, the TMFSS model is more conducive to the optimisation of acoustic parameters.

The ability to modulate permeability by tuning bubble size and spacing (Figures 11 and 12) offers a high degree of control. The finding that larger bubbles dominate the dynamics suggests that carefully engineered bubble size distributions could be used to target specific vascular regions. The existence of an optimal interbubble distance for shear stress generation is critical for designing microbubble dosing and administration strategies to ensure they are in close enough proximity (Song 2021; Huang et al. 2024).

The incorporation of the shear-thinning Carreau-Yasuda model revealed the complex, nonlinear feedback between flow fields and viscosity (Figures 6 and 7). The resulting damping effect on bubble oscillations and the altered stress profiles (Figure 8a) demonstrate that assuming constant viscosity oversimplifies the system dynamics. This nonlinearity suggests that the endothelial response itself may be adaptive, with pores potentially opening and closing repeatedly before irreversible damage occurs, a phenomenon that warrants further biological investigation.

Although ultrasound-assisted TMFSS significantly improves vascular permeability, creating critical structural conditions for paracellular drug transport, this study has several limitations. The specific transport mechanisms of hydrophilic macromolecular drugs (such as peptides and nucleic acid drugs) via paracellular transport have not been fully characterised. Furthermore, the model assumes pre-fixed bubble positions, neglecting the translational motion and coalescence of microbubbles in a flowing blood stream. Future work should focus on developing 3D models that include the dynamic motion of microbubbles in flow and couple the mechanical stimuli with predictive models of endothelial bioresponse. Ultimately, a deeper understanding of the TMFSS will enable the design of precise, localised, and safe sonoporation strategies for delivering therapeutics to the brain and other protected tissues.

## 5 Conclusions

In this study, a novel coupled rheology-based two-microbubble fluid‒solid interaction (TMFSS) model was developed. The simulations demonstrate that synergistic cavitation within the dual-microbubble system significantly enhances (>2-fold compared with single-microbubble cavitation) vascular endothelial permeability. By incorporating shear-rate-dependent non-Newtonian blood rheology into cavitation dynamics and systematically optimising key parameters—acoustic pressure, frequency, microbubble size, and interbubble spacing—our model enables the maximisation of permeabilization efficacy while minimising the risk of cavitation-induced vascular damage. This work establishes a computational framework for the precise spatiotemporal control of intravascular drug transport, offering significant potential for advancing targeted, precision drug delivery strategies, particularly for overcoming endothelial barriers in diseases such as brain disorders.

## Data Availability

All original contributions presented in this study are included in the article. Further inquiries can be directed to the corresponding author(s).
